# Immunomodulation by mesenchymal stem cells in treating human autoimmune disease-associated lung fibrosis

**DOI:** 10.1186/s13287-016-0319-y

**Published:** 2016-04-23

**Authors:** Ming Liu, Xiansheng Zeng, Junli Wang, Zhiping Fu, Jinsong Wang, Muyun Liu, Dunqiang Ren, Baodan Yu, Lixia Zheng, Xiang Hu, Wei Shi, Jun Xu

**Affiliations:** State Key Laboratory of Respiratory Diseases, Guangzhou Institute of Respiratory Diseases, The First Affiliated Hospital of Guangzhou Medical University, Guangzhou Medical University, Guangzhou, P. R. China; Developmental Biology and Regenerative Medicine Program, Department of Surgery, The Saban Research Institute of Children’s Hospital Los Angeles, University of Southern California Keck School of Medicine, Los Angeles, CA USA; Department of Respiratory Medicine, Xiangyang Central Hospital, Xiangyang, Hubei province P. R. China; Shenzhen Beike Cell Engineering Research Institute, Shenzhen, P. R. China

**Keywords:** Mesenchymal stem cells, Regulatory T cells, Natural killer T cells, Lung fibrosis in autoimmunity, TGF-β1, IL-6, IP-10

## Abstract

**Background:**

Interstitial pneumonia in connective tissue diseases (CTD-IP) featuring inflammation and fibrosis is a leading cause of death in CTD-IP patients. The related autoimmune lung injury and disturbed self-healing process make conventional anti-inflammatory drugs ineffective. Equipped with unique immunoregulatory and regenerative properties, mesenchymal stem cells (MSCs) may represent a promising therapeutic agent in CTD-IP. In this study, we aim to define the immunopathology involved in pulmonary exacerbation during autoimmunity and to determine the potential of MSCs in correcting these disorders.

**Methods:**

Lung and blood specimens, bronchoalveolar lavage fluid cells collected from CTD-IP patients, and human primary lung fibroblasts (HLFs) from patients pathologically diagnosed with usual interstitial pneumonia (UIP) and healthy controls were analyzed by histology, flow cytometry and molecular biology. T cell subsets involved in the process of CTD-IP were defined, while the regulatory functions of MSCs isolated from the bone marrow of normal individuals (HBMSCs) on cytotoxic T cells and CTD-UIP HLFs were investigated in vitro.

**Results:**

Higher frequencies of cytotoxic T cells were observed in the lung and peripheral blood of CTD-IP patients, accompanied with a reduced regulatory T cell (Treg) level. CTD-UIP HLFs secreted proinflammatory cytokines in combination with upregulation of α-smooth muscle actin (α-SMA). The addition of HBMSCs in vitro increased Tregs concomitant with reduced cytotoxic T cells in an experimental cell model with dominant cytotoxic T cells, and promoted Tregs expansion in T cell subsets from patients with idiopathic pulmonary fibrosis (IPF). HBMSCs also significantly decreased proinflammatory chemokine/cytokine expression, and blocked α-SMA activation in CTD-UIP HLFs through a TGF-β1-mediated mechanism, which modulates excessive IL-6/STAT3 signaling leading to IP-10 expression. MSCs secreting a higher level of TGF-β1 appear to have an optimal anti-fibrotic efficacy in BLM-induced pulmonary fibrosis in mice.

**Conclusions:**

Impairment of TGF-β signal transduction relevant to a persistent IL-6/STAT3 transcriptional activation contributes to reduction of Treg differentiation in CTD-IP and to myofibroblast differentiation in CTD-UIP HLFs. HBMSCs can sensitize TGF-β1 downstream signal transduction that regulates IL-6/STAT3 activation, thereby stimulating Treg expansion and facilitating anti-fibrotic IP-10 production. This may in turn block progression of lung fibrosis in autoimmunity.

**Electronic supplementary material:**

The online version of this article (doi:10.1186/s13287-016-0319-y) contains supplementary material, which is available to authorized users.

## Background

Interstitial pneumonia (IP) is a heterogeneous group of lung parenchymal disorders, with common pathological features of inflammation and/or fibrosis. Fibrosis in IP patients is often irreversible, resulting in significant morbidity and mortality [1]. IP can be idiopathic (idiopathic pulmonary fibrosis, IPF) or secondary to exposure to a variety of harmful environmental factors. Although the pathogenesis of IP is not yet clear, a subgroup of IP is associated with connective tissue diseases (CTD-IP), including multiple sclerosis, rheumatoid arthritis (RA) and polymyositis/dermatomyositis (PM/DM) [2, 3]. The pathological features of CTD-IP can be nonspecific IP (NSIP), usual IP (UIP), cryptogenic organizing pneumonia (COP), acute interstitial pneumonia and diffuse alveolar damage. The frequency of IP in these CTDs varies, ranging from 20 % to more than 50 % and presenting either before or after these CTDs are diagnosed. More importantly, IP pathologically diagnosed UIP, in particular, is a leading cause of death in these patients. There is no effective treatment currently available, although immunosuppressive and anti-inflammatory drugs, such as corticosteroids, have been widely used.

Recent studies have reported that local and systemic immune activation and impairment of immunological tolerance were detected in CTD-IP patients [4–10]. For example, RA patients had a greater number of CD4-positive T cells in the bronchoalveolar lavage (BAL) fluid than IPF patients [11]. Increased autoantibodies against topoisomerase and Jo-1 were strongly associated with development of IP in multiple sclerosis and PM/DM patients, respectively [12, 13]. Abnormalities in T cells, including T regulatory cells (Tregs) in autoimmunity may play an important role in pulmonary fibrosis in CTD-IP [9]. However, it is still unclear which subsets of immune cells are involved in pulmonary fibrosis and how they affect the development of disease [10, 14], although it is conceivable that dysregulation of the immune system may be an important factor contributing to CTD-IP. Therefore, the characterization of these immunological changes at the molecular and cellular levels in CTD-IP patients and the discovery of novel approaches to correcting these changes will be critical for treating CTD-IP in the future [15, 16].

The immunomodulatory properties of mesenchymal stem cells (MSCs) have recently caused excitement for investigators examining their potential therapeutic application in a variety of immune disorder diseases [17, 18]. MSCs have been tested in rodent models to treat diseases where immunodysregulation is thought to be the main pathogenic mechanism. It has been shown that MSCs can reverse autoimmune response disorder by modulating multiple subsets of immune cells [19]. In addition, their pluripotent nature may also benefit CTD-IP patients by directly or indirectly promoting alveolar repair [20]. Recent studies have demonstrated the capability of MSCs to inhibit bleomycin-induced pneumonitis and fibrosis in a mouse model [21]. However, it has been argued that bleomycin-induced pulmonary fibrosis in a mouse system does not reflect all of the immunological mechanisms involved in human CTD-IP or IPF. Herein, we have characterized the main features of the immune disorder in CTD-IP patients at the active stage of the disease. We found that persistent activation of an innate immune response by high frequency natural killer T cells (NKTs) in the circulation and lung was linked to the promotion of CTD-IP, where the pulmonary fibroblasts obtained a myofibroblast phenotype that persisted in the culture. Using an experimental NKT-peripheral blood mononuclear cell (PBMCs) model in vitro and isolated primary lung fibroblasts from CTD-IP patients pathologically diagnosed with usual interstitial pneumonia (UIP), we demonstrate that MSCs have great potential to inhibit fibrotic development in CTD-IP by sensitization of attenuated TGF-β1 downstream signal, which, in turn, exerts anti-inflammatory and anti-fibrotic effects.

## Methods

### Subjects

A total of 28 CTD- IP patients (12 patients with rheumatoid arthritis (RA)-IP and 16 patients with polymyositis/dermatomyositis (PM/DM)-IP) who were hospitalized in the affiliated Hospital of Guangzhou Medical University from January 2010 to March 2013 were enrolled in this study, and 23 healthy volunteers were used as control subjects. All patients met the interstitial lung disease and connective tissue disorder-related criteria [22]. The study protocol was approved by the Ethics Committee of the First Affiliated Hospital of Guangzhou Medical University, and informed consent was obtained from all patients and control subjects. Flow cytometry was performed on peripheral blood (PB) and bronchoalveolar lavage fluid (BAL) samples. Baseline characteristics of the studied patients are shown in Tables 1 and 2.

**Table 1:** Subject characteristics (*n* = 51)^a^

Variable	Subjects	Control (N, %)	CTD-IP (N, %)	*P*-Value
Total	51	23 (45.1)	28 (54.9)	
Age				0.723
≥50 years	28	12 (42.9)	16 (57.1)	
<50 years	23	11 (47.8)	12 (52.2)	
Gender				0.654
Male	22	10 (45.5)	12 (54.5)	
Female	29	13 (44.8)	16 (55.2)	

^a^Data are expressed as number (percentage)

**Table 2:** Clinical summary of patients with CTD-IP (*n* = 28)^a^

Characteristics	Data
Age	52 (25–78)
Male gender	12
Female gender	16
Smoking history	
Never	15
Former	6
Current	7
CTD history	28
DM/PM	16
RA	12
Abnormal RF/CRP level	25
Time since CTD diagnosis, yr	4.10 (2–11)
Time since CTD-IP diagnosis, yr	3.48 (0.5–10)

*Abbreviations*: *CTD* connective tissue disease, *DM/PM* dermatomyositis/polymyositis, *RA* rheumatoid arthritis, *RF/CRP* rheumatoid factor/C-reactive protein

^a^Data are expressed as the median (interquartile range) or number

### Lung histology and immunohistochemistry

Human lung paraffin sections prepared from lung biopsy specimens of the enrolled patients were stained with hematoxylin and eosin (H&E) for histopathology. Collagen was stained using the Masson trichrome method (Maixin-bio, China). Immunostaining was performed as previously described [23], using antibodies against α smooth muscle actin (α-SMA) (1:400, A2547, Sigma, St Louis, MO, USA) and CD3 (1:100, ab5690, Abcam, Cambridge, UK).

### Culture of human lung fibroblasts

Primary human lung fibroblasts (HLFs) were prepared from the lung biopsies of CTD-IP patients (*n* = 4) pathologically diagnosed with usual interstitial pneumonia (UIP). Primary normal human lung fibroblasts (NHLFs) derived from normal tissue areas of surgical lobectomy specimens taken from patients with lung cancer were used as a negative control. Cell culture was performed according to the Primary Lung Fibroblast Culture protocol given in Additional file 1: Methods.

After serum starvation for 24 h, NHLFs were treated with TGF-β1, IL-6 alone or in combination and cytomix (a mixture of TGF-β1, IFN-γ, and IL-1β (all from R&D Systems)) for 48 hours.

### Preparation of human bone marrow mesenchymal stem cells

Human bone marrow mesenchymal stem cells (HBMSCs) were isolated from the bone marrow of normal individuals undergoing bone marrow harvest for allogeneic bone marrow transplantation. Informed consent was obtained and the study protocol was approved by the Ethics Committee of the First Affiliated Hospital of Guangzhou Medical University. MSCs derived from umbilical cord (UC) were also isolated. MSCs culture and verification were performed as described in the figure in Additional file 2.

### Generation of natural killer T cell-peripheral blood mononuclear cells

Peripheral blood was provided by the Guangzhou Blood Center after approval was given by the Department of Health of Guangdong Province. The generation and identification of natural killer T cell-peripheral blood mononuclear cells (NKT-PBMCs) were performed as described in the figure in Additional file 3.

### Cell co-culture

#### Co-culture of HBMSCs and PBMCs

Cultured HBMSCs or NHLF were added to NKT-PBMCs, PBMC from healthy controls, and IPF patients (*n* = 12) at a 1:20 ratio for 24 or 48 hours. After that, the treated PBMCs were collected for flow cytometry analysis.

#### Co-culture of HBMSCs and HLFs

HBMSCs were co-cultured with NHLF or CTD-UIP-HLF at a 1:1 ratio using Transwell chambers (Corning, Tewksbury, MA, USA). HBMSCs were plated into the upper chamber, and NHLF or CTD-UIP-HLFs were plated into the lower chamber. CTD-UIP HLF were treated with MSC or TGF-β1 in the absence and presence of neutralizing antibody for either human IP-10 (2 μg/ml) (C) or human TGF-β1(1 μg/ml). The entire culture system was maintained for 48 hours in an incubator containing 5 % CO_2_, then NHLFs or CTD-IP-HLFs were lysed for western blot analysis.

### Flow cytometry

NKT-PBMCs and whole peripheral blood samples from healthy controls and CTD-IP patients were stained with the following antibodies: CD3-FITC, CD56-PE, CD127-PE, CD45-ECD, CD4-FITC, CD25-PC5, CD4-FITC/CD8-PE/CD3-PC5, FOXP3–PE, and appropriate isotype controls (Beckman Coulter, Indianapolis, IN, USA). Staining was performed according to the manufacturer’s instructions.

### Western blot

Protein expression and phosphorylation were determined by western blot, as previously described [23]. Briefly, cells were lysed in radioimmunoprecipitation (RIPA) buffer, then subjected to polyacrylamide gel electrophoresis and incubated with primary antibodies at 4 °C overnight, then incubated with secondary antibodies and developed by chemiluminescence reaction (Pierce). Digital chemiluminescent images were obtained and quantified with a Kodak image station 4000R system. Primary antibodies used in this study were anti-fibronectin (Santa Cruz Biotechnology), anti-vimentin (Santa Cruz Biotechnology), anti-α-SMA antibody (Sigma), anti-STAT3, anti-phosphorylated STAT3, and anti-phosphorylated Smad3 (Cell Signaling).

### ELISA and liquid microarray assay

Human TGF-β1 secreted from the cultured cells into medium was measured using an ELISA kit (R&D Systems, Minneapolis, MN, USA). The levels of the cytokines interferon γ (IFN-γ), tumor necrosis factor α (TNF-α), interleukin 8 (IL-8), IL-6, macrophage inflammatory protein-1α (MIP-1α), monocyte chemoattractant protein-1 (MCP-1), MCP-3, IFN-γ-inducible protein 10 (IP-10), and vascular cell adhesion molecule-1 (VCAM-1) were determined by a liquid microarray assay using Luminex technology (Merck Millipore, Billerica, MA, USA).

### Animals and experimental groups

C57BL/6 mice aged 8 weeks (*n* = 80) (Guangdong Medical Laboratory Animal Center, China) were randomly divided into four groups: control group (mice treated with saline solution), BLM group (mice challenged with BLM), and MSC treatment groups (treatment of mice with the supernatant from human MSC-BM or MSC-UC). A BLM-induced lung fibrosis mouse model was induced as described [24] by intratracheal addition of 3 U/kg body weight BLM (Nippon Kayaku Co., Ltd. Japan). Supernatants harvested from MSCs (1 × 10^6^) culture were concentrated and intratracheally added to the mouse model 48 hours after BLM administration. Survival rates and lung histological sections were analyzed in mice 21 days after BLM exposure. All animal study protocols were reviewed and approved by the University Committee on Use and Care of Animals at Guangzhou Medical University.

### Statistical analysis

All data are expressed as the mean ± SD. Statistical differences between different groups were evaluated using the Student’s *t* test. All analysis was performed using the SPSS 10.0 software package (SPSS, Chicago, IL, USA). A *P*-value of *P* ≤ 0.05 was considered as statistically significant.

## Results

### Pulmonary interstitial inflammation and fibrosis in CTD-IP patients are accompanied by significantly increased numbers of NKT cells

**Fig. 1:**
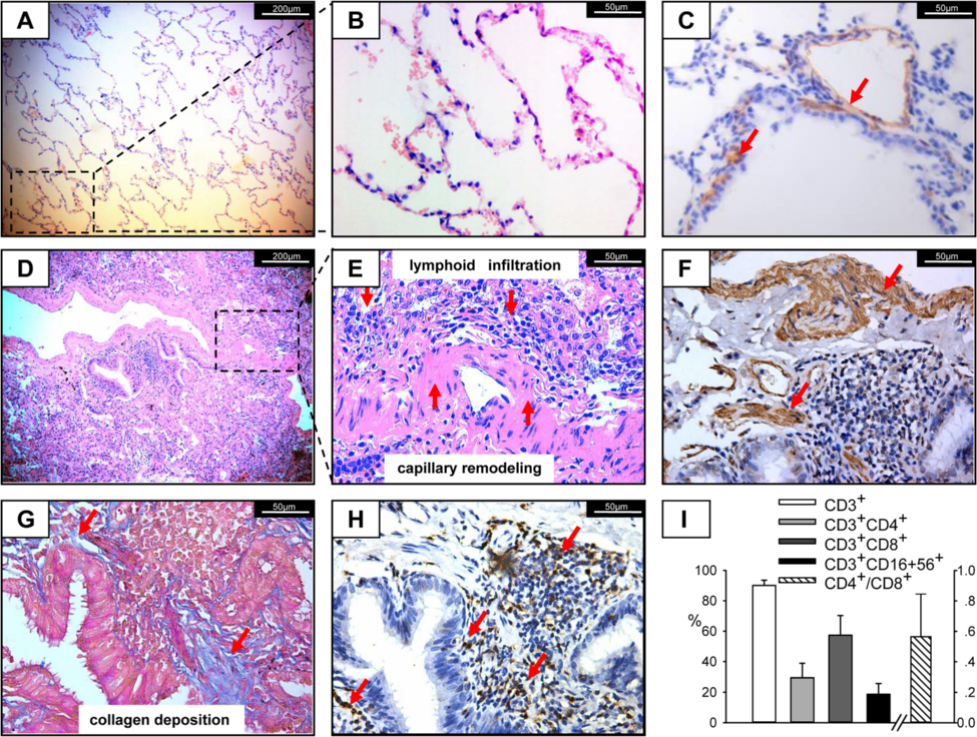
The frequency of NKT cells is increased in the lung of CTD-IP patients. Representative hematoxylin and eosin (HE) stained lung sections from healthy control (**a**, **b**) and enrolled CTD-IP patients (*n* = 6) (**d**, **e**) showing areas of sub-acute alveolar damage accompanied by capillary remodeling (**d**, **e**) and lymphoid follicle formation (**d**, **e**) in CTD-IP. Lung sections stained with Masson trichrome (MT) and immunostaining showed increased collagen deposition (blue, **g**), combined with enhanced expression of α-SMA (brown, **f**) in capillaries and interstitial cells compared with healthy control (brown, **c**). Positive CD3 immunostaining was located in the lymphoid follicles (brown, **h**). The *arrows* indicate myofibroblast infiltration with α-SMA-positive staining or T cells with CD3-positive staining. (**a**, **d**) 100× magnification, (**b**), (**c**), (**e**) to (**h**) 400× magnification. **i** Flow cytometric analysis of BALF cells, percentage of CD3^+^, CD3^+^ CD4^+^, CD3^+^ CD8^+^, CD3^+^ CD56^+^ cells gating on leucocytes and CD8^+^/CD4^+^ are presented, and the means ± SD of six cases are shown. *α-SMA* α-smooth muscle actin, *BALF* bronchoalveolar lavage fluid

The histopathology of lung tissue biopsy specimens from healthy controls (Fig. 1a, b) and enrolled CTD-IP patients (*n* = 6) was examined after H&E staining (Fig. 1d, e). Sub-acute alveolar damage accompanied by patchy alveolar pneumocyte hyperplasia and capillary remodeling was consistently observed (Fig. 1d, e). Moreover, diffuse chronic inflammation and fibrosis were detected in lung parenchyma, resulting in thickened interstitial spaces with accumulation of myofibroblasts and extracellular matrix, especially collagen (Fig. 1d, f and g). By immunostaining, the majority of infiltrated CD3^+^ T cells were detected in the airway and pulmonary interstitial spaces, as well as lymphoid follicles (Fig. 1h). Furthermore, analyses of inflammatory cells in patients’ BAL fluids by flow cytometry showed that more than 85 % of the leucocytes were CD3^+^ T cells, including CD8^+^ T cells, CD3^+^ CD56^+^ NKT cells and CD4^+^ T cells (Fig. 1i).

### Correlations of the aberrant T subsets and cytokine profiles in the systemic circulation for the impaired pulmonary function

**Fig. 2:**
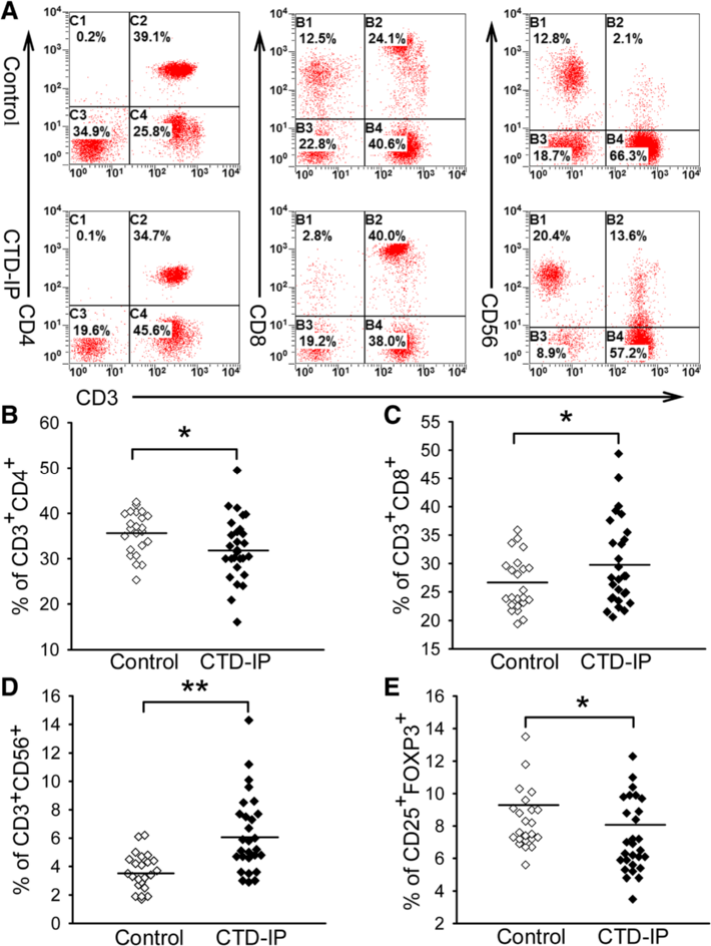
The frequency of NKT cells in the peripheral blood of CTD-IP patients is increased accompanied by reduction of Tregs. **a** Gating on lymphocytes, flow cytometric analysis of CD3^+^ CD4^+^ T cells, CD3^+^ CD8^+^ T cells and CD3^+^ CD56^+^ cells in the peripheral blood of healthy controls and patients with CTD-IP. **b**–**e**) scatter plots of the percentage of CD3^+^ CD4^+^ T cells, CD3^+^ CD8^+^ T cells and CD3^+^ CD56^+^ cells gating on lymphocytes, and CD25^+^ FOXP3^+^ cells gating on CD4^+^ cells in the peripheral blood of healthy controls (*n* = 23) and patients with CTD-IP (*n* = 28). * *P* < 0.05, ** *P* < 0.01 for all comparisons between CTD-IP and control. *NKT* natural killer T cells, *CTD-IP* interstitial pneumonia in connective tissue disease, *Tregs* regulatory T cells

We next determined if the altered lymphocyte profiles also occurred in the systemic circulation of the CTD-IP patients using flow cytometry (Fig. 2). By comparing CTD-IP patients (*n* = 28) with the normal control group (*n* = 23), we found that CD3^+^ CD56^+^ NKT-like cells were significantly increased in the peripheral blood of CTD-IP patients (Fig. 2a and d, 6.26 ± 2.74 % in CTD-IP vs. 3.65 ± 1.27 % in controls, *P* = 0.003). Meanwhile, elevation of CD3^+^ CD8^+^ cells (29.96 ± 7.62 % in CTD-IP vs. 26.40 ± 4.78 % in control, *P* = 0.048) and reduction of CD3^+^ CD4^+^ cells (32.23 ± 6.95 % in CTD-IP vs. 35.71 ± 4.69 % in control, *P* = 0.046) were also detected (Fig. 2a–c). In addition, a reduced number of CD4^+^ CD25^+^ FOXP3^+^ Tregs was observed in the CTD-IP patients compared with normal controls (7.32 ± 2.21 % in CTD-IP vs. 8.36 ± 1.81 % in control, *P* = 0.035), as shown in Fig. 2e.

**Fig. 3:**
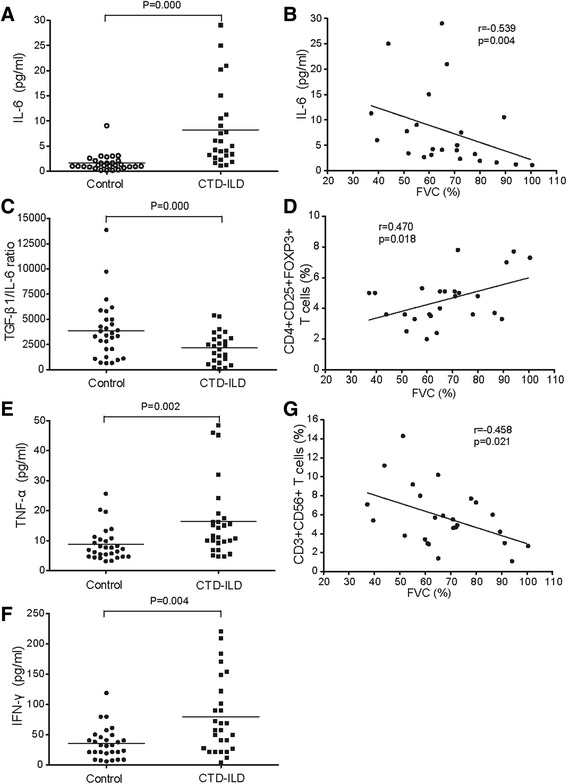
Correlations of the altered T cell subsets and cytokine profiles with pulmonary functions in the patients with CTD-ILD. **a**, **c**, **e**, **f**) The plasma levels of IL-6, TGF-β/IL-6 ratio, TNF-α, and IFN-γ in the CTD-ILD patients who had not received corticosteroid therapy (*n* = 27) and healthy control subjects (*n* = 29). Each point represents one person. The median value for each group is indicated by a *horizontal line*. **b**, **d**, **g** Correlations of forced vital capacity (FVC) with the altered T cell subsets and cytokines. **b**, **d** Correlations of the increased plasma IL-6 level or declining peripheral blood regulatory T cells (Tregs) with worsening FVC. **g** Correlations of the elevation of CD3^+^CD56^+^ NKT cells with the reduction of FVC. P values were obtained by Pearson’s test. *IL-6* interleukin-6, *TGF-β* transforming growth factor-β, *TNF-α* tumor necrosis factor α, *IFN-γ* interferon γ, *NKT* natural killer T cells, *FVC* forced vital capacity

We then asked if the cytokine profile in the patients’ peripheral blood exhibited corresponding changes, which were involved in pulmonary fibrotic development in autoimmunity. As predicted, we detected significantly increased production of pro-inflammatory/fibrotic cytokines, including IL-6, IFN-γ, TNFα, and TGF-β1 in CTD-ILD patients compared with that in normal controls. The augmentation of IL-6 level, rather than TGF-β1, has a negative correlation with a lung function parameter, forced vital capacity (FVC) (Fig. 3a, b), corresponding to a decreased TGF-β1/IL-6 ratio relevant to down-regulation in the Tregs level, which is closely correlated with the declining FVC (Fig. 3c, d). High levels of TNF-α and IFN-γ in circulation associated with an increase in the NKT cell level, was also responsible for reduced FVC (Fig. 3e, f, g).

### The autoimmune inflammatory microenvironment induces pulmonary myofibroblast differentiation in CTD-IP

**Fig. 4:**
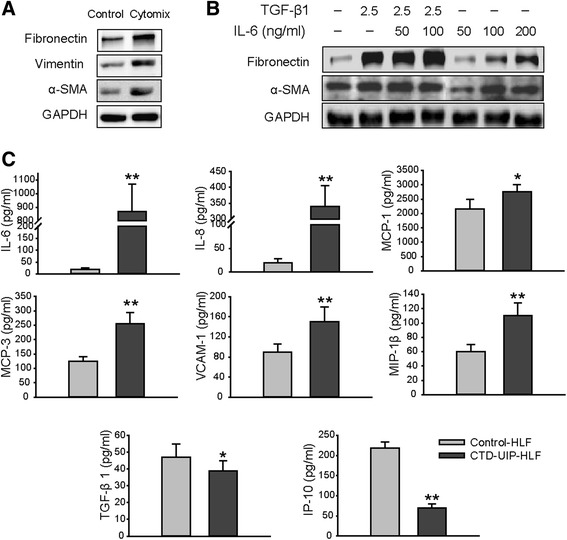
HLFs differentiation towards myofibroblast after exposure to inflammatory cytomix is linked to the characteristic feature of CTD-UIP HLF’s phenotype. **a**, **b** Western blot was performed on normal HLFs treated with cytomix (a mixture of cytokines) (**a**) or TGF-β/IL-6 (**b**) for examination of expression of α-SMA, vimentin, and fibronectin. Data are representative of three independent experiments. **c** Levels of cytokines and chemokines were measured in culture supernatants of human lung fibroblasts (HLF) from patients with CTD-UIP (CTD-UIP HLF) and normal controls (NHLF) using Luminex multiplex technology. Data are representative of two independent experiments. Significance of difference between independent groups of data (mean ± SD) was analyzed by Student’s *t* test (two-tailed). * *P* < 0.05, ** *P* < 0.01 for all comparisons between CTD-IP-HLF and NHLF. *CTR-UIP-HLF* HLF isolated from the lung tissues pathologically diagnosed with UIP in CTD-IP patients, *NHLF* normal human lung fibroblasts, *TGF-β* transforming growth factor-β, *IL-6* interleukin-6, *α-SMA* α-smooth muscle actin

We next tested the impact of a mixture of cytokines (cytomix), which have been demonstrated to be significantly increased in peripheral blood in CTD-IP patients, on myofibroblast development. We detected a myofibroblast differentiation with marked over expression of αSMA, vimentin, and fibronectin in the normal lung fibroblasts (NHLFs) after exposure to cytomix (Fig. 4a). Low dosage IL-6 addition enhances TGF-β1-induced myofibroblast activation, whereas administration of IL-6 alone can also induce myofibroblast differentiation in a concentration-dependent manner (Fig. 4b).

We observed significantly elevated release of proinflammatory cytokines, including IL-6, IL-8, MIP-1α, MCP-1, MCP-3, VCAM-1 and MIP-1β, from lung fibroblasts (HLFs) derived from CTD-IP patients (*n* = 4) with pathologically diagnosed usual interstitial pneumonia (UIP) (CTD-UIP HLFs), compared with NHLFs (Fig. 4c, *P* < 0.05 or *P* < 0.01). In contrast, production of the anti-fibrotic cytokine IP-10 was significantly reduced in CTD-UIP HLFs (Fig. 4c, *P* < 0.01). Surprisingly, TGF-β1, which is an anti-inflammatory, but profibrotic factor, was slightly reduced in the UIP-HLFs. The combined anti-fibrotic effect as measured by the ratio of IP-10 to TGF-β1 was also decreased (4.58 in NHLFs vs. 2.09 in CTD-UIP HLFs).

### HBMSCs induce Tregs expansion in either NKT-PBMCs model or PBMCs isolated from IPF patients

Given that human MSCs are emerging as a therapeutic modality in various inflammatory diseases owing to their immunomodulatory properties [25], we examined the regulatory effect of MSCs on cytotoxic NKT cell induction in an established in vitro system, in which high frequency NKT cells can be induced from fresh peripheral blood mononuclear cells (PBMCs) of healthy volunteers by cytokine treatment [26]. In the present study, CD3^+^ CD56^+^ NKT cells were markedly induced (27.3 ± 6.3 %) from PBMCs after cytokine treatment in vitro, compared with less than 5 % of NKT cells in untreated PBMCs. Furthermore, another type of cytotoxic T cell, CD3^+^ CD8^+^ T cells, increased 2-fold, while CD3^+^ CD4^+^ T cells had a 1-fold reduction in the treated PBMCs compared with untreated PBMCs (Additional file 4: Figure S3). Thus, the alterations of T cell subtypes in cytokine-treated PBMCs in vitro mimics the changes detected in peripheral blood of CTD-IP patients.

**Fig. 5:**
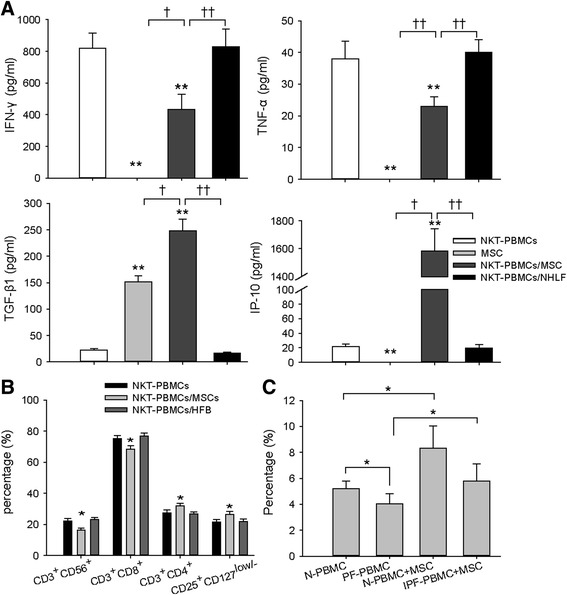
Immunomodulatory effects of human bone marrow MSCs on aberrant T subsets and cytokines profile. **a**, **b** NKT-PBMCs were co-cultured with human MSCs or human fibroblasts at a 20:1 ratio of NKT-PBMCs to human MSCs or NHLF prior to cytokines test in the supernatants (**a**) and flow cytometric analysis (**b**) for each group. Triplicate wells were prepared for each group. **a** TNF-α, IFN-γ, TGF-β1, and IP-10 levels in the supernatants of NKT-PBMCs, MSCs, and NKT-PBMCs co-cultured with human bone marrow MSCs or NHLF. ** Significantly different from the NKT-PBMCs group, *P* < 0.01. † *P* < 0.05, †† *P* < 0.01, compared to MSCs or NKT-PBMCs co-cultured with NHLF. Data represent the means ± SD from three independent experiments. **b** Flow cytometric analysis of CD3^+^ CD56^+^ cells, CD3^+^ CD8^+^ cells, CD3^+^ CD4^+^ cells gating on CD45^+^ cells, and CD25^+^ CD127^(Low/-)^ Treg cells gating on CD4^+^ cells, of either NKT-PBMCs (NKT-PBMCs) or NKT- PBMCs co-cultured with human bone MSCs (NKT-PBMCs/MSC, or co-cultured with NHLF (NKT-PBMCs/NHLF). *P < 0.05 for comparisons between NKT-PBMCs/MSC and NKT-PBMCs/NHLF or NKT-PBMCs. Data represent the means ± SD from three independent experiments. **c** CD25^+^ FOXP3^+^ Treg cells gating on CD4^+^ cells in the PBMCs of healthy controls and IPF patients (*n* = 12) before and after being co-cultured with MSCs or human fibroblasts. Data represent the means ± SD. *P < 0.05. *MSCs* mesenchymal stem cells, *NKT* natural killer T cells, *PBMCs* peripheral blood mononuclear cells, *NHLF* normal human lung fibroblasts, *TNF-α* tumor necrosis factor-α, *IFN-γ* interferon γ, *TGF-β* transforming growth factor-β, *IP-10* interferon γ-induced protein 10

We then investigated the role of human MSCs in modulating T cell subtypes in vitro using the system described above. As shown in Fig. 5b and Additional file 4: Figure S3, co-culture of HBMSCs with NKT-PBMCs in the presence of NKT-inducing agents resulted in a significant reduction in NKT cells from 20.33 ± 1.05 % in the MSC-free control to 15.17 ± 1.75 % with MSC treatment (*P* < 0.05), and caused a decrease of CD3^+^ CD8^+^ T cell induction, but up-regulated CD3^+^CD4^+^ and CD4^+^CD25^+^ CD127^(low/-)^/foxp3^+^ T cells, accompanied by significantly diminished IFN-γ and TNF-α, and elevated TGF-β1 and IP-10 in the co-culture supernatants (Fig. 5a*P* < 0.01). A high level of TGF-β1 was also detected in the culture of HBMSCs alone. The specificity of the MSC’s effect was further verified by co-culturing NHLFs with NKT-PBMCs. No effect on NKT cell induction was observed by co-culturing PBMC with NHLFs. Likewise, we confirmed that HBMSCs have the ability to induce Tregs expansion in the IPF patients’ PBMCs where there was a suppressed Tregs growth compared to normal controls (Fig. 5c).

### HBMSCs inhibit the proinflammatory and profibrotic properties of UIP-HLFs through regulation of excessive IL-6 signaling activation

**Fig. 6:**
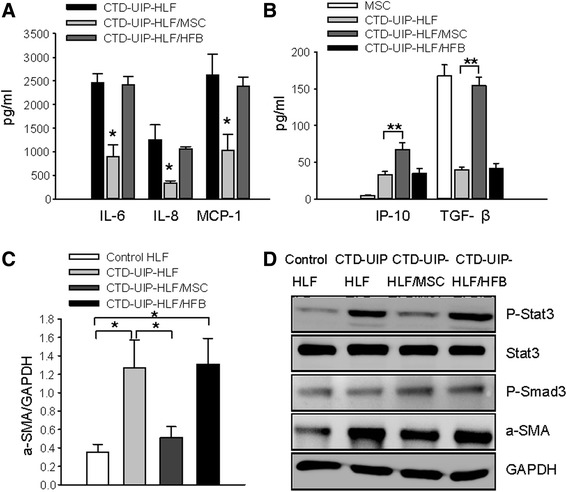
Immunomodulatory effects of human bone marrow MSCs on CTD-UIP HLFs. **a** IL-6, IL-8, and MCP-1 levels in cultures of CTD-UIP HLF and CTD-UIP HLF pre-treated with either MSCs or NHLF. Triplicate wells were prepared for each group. Data represent the means ± SD from four independent experiments. * Significantly different from CTD-IP-HLF, *P* < 0.05. **b** IP-10 and TGF-β1 levels in cultures of MSCs, CTD-UIP HLF, and CTD-UIP HLF pre-treated with either MSCs or NHLF. Triplicate wells were prepared for each group. Data represent the means ± SD from four independent experiments. * or ** significantly different from the MSC group, *P* < 0.05 or *P* < 0.01 respectively. † *P* < 0.05, †† *P* < 0.01, compared to CTD-UIP HLF pre-treated with NHLF or CTD-UIP HLF without the pretreatment. **c,** **d** Western blot analysis was performed to assess α-SMA expression and signaling pathways (stat3 and smad3) in NHLF, CTD-UIP HLF, and CTD-UIP HLF pre-treated with either MSCs or NHLF. GAPDH was used as a loading control. Representative blots from three replicates are shown (**d**). Quantification of α-SMA expression (**c**). * Significantly different from the NHLF group with *P* < 0.05. † *P* < 0.05, compared to CTD-UIP HLF pre-treated with NHLF or CTD-UIP HLF without the pretreatment. *MSCs* mesenchymal stem cells, *CTD-UIP-HLF* HLF isolated from lung tissues pathologically diagnosed with UIP in CTD-IP patients, *HLF* human lung fibroblasts, *NHLF* normal human lung fibroblasts, *IP-10* interferon γ-induced protein 10, *TGF-β1* transforming growth factor-β1, α-SMA α-smooth muscle actin

To investigate the role of human MSCs in the modulation of CTD-UIP HLFs, we performed a co-culture of HBMSCs and CTD-UIP HLFs. Similarly, we detected a high level of TGF-β1 in the supernatant either of the co-culture system or HBMSC alone (Fig. 6b, *P* < 0.01), concomitant with marked suppression of IL-6, IL-8, and MCP-1 (Fig. 6a, *P* < 0.05) and a significantly elevated IP-10 secretion in comparison to the co-culture of CTD-UIP HLFs with NHLFs. Co-culture of CTD-UIP HLFs with HBMSCs, but not NHLFs, attenuated α-SMA hyperexpression in the UIP HLFs (Fig. 6c, *P* < 0.05). Furthermore, we found that hyperphosphorylation of STAT3 attributed to excessive IL-6 secretion in CTD-UIP HLFs was significantly blocked by HBMSC treatment, whereas phosphorylation of Smad3 was slightly upregulated (Fig. 6d).

### TGF-β1 hypersecretion in HBMSCs rescues attenuated TGF-β1 downstream signal transduction for induction of expression of anti-fibrotic chemokine IP 10

**Fig. 7:**
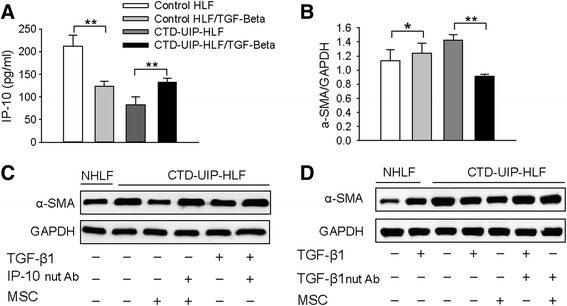
Suppression of the myofibroblast phenotype in CTD-UIP HLF through the activation of attenuated TGF-β1 signaling and subsequent IP-10 induction. **a**, **b** IP-10 levels (**a**) and western blot analysis of α-SMA expression (**b**) in NHLF and CTD-UIP HLF in the absence or presence of TGF-β1. Data are representative of three independent experiments. Representative blots from three replicates are shown. Quantification of α-SMA expression by densitometric analysis was performed using Gel-Pro software. * *P* < 0.05, ** *P* < 0.01. **c**, **d** Representative western blot for α-SMA expression in CTD-UIP HLF treated with MSC or TGF-β1 in the absence and presence of neutralizing antibody for either human IP-10 (2 ug/ml) (**c**), or human TGF-β1(1 ug/ml) (**d**). GAPDH was used as a loading control. Representative blots from three replicates are shown. *CTD-UIP-HLF* HLF isolated from lung tissues pathologically diagnosed with UIP in CTD-IP patients, *HLF* human lung fibroblasts, *TGF-β1* transforming growth factor-β1, *IP-10* interferon γ-induced protein 10, α-SMA α-smooth muscle actin, *NHLF* normal human lung fibroblasts

As TGF-β1 is a profibrotic growth factor that stimulates α-SMA expression and myofibroblast differentiation, we investigated the paradox that TGF-β1 hypersecretion in MSC resulted in an increased level of IP-10 in UIP-HLF and concurrently reduced α-SMA expression. In NHLFs, addition of TGF-β1 elevated α-SMA expression (Fig. 7, *P* < 0.05), accompanied by suppression of IP-10 production (Fig. 7, *P* < 0.01). However, in CTD-UIP HLFs, addition of TGF-β1 significantly increased IP-10 secretion and down-regulated α-SMA expression (Fig. 7, *P* < 0.01), suggesting that UIP-HLFs have an opposite response to TGF-β1 stimulation compared with NHLFs, and that the negative regulatory effect of IP-10 on α-SMA expression may be downstream of the TGF-β1 pathway.

To elucidate the role of IP-10 elevation induced by TGF-β1-expressing MSCs in modulating UIP-HLFs, a human IP-10-neutralizing antibody (R&D Systems, AF-266-NA) was administrated to HBMSCs, prior to co-culture with CTD-UIP HLFs for 48 hours. The western blot data showed that IP-10 neutralization partly reversed the suppression of α-SMA up-regulation caused by MSC treatment. Similarly, addition of IP-10-neutralizing antibody blocked the efficacy of TGF-β1 administration on CTD-UIP HLFs (Fig. 7c). A consistent result was also observed in HBMSCs treated with TGF-β1-neutralizing antibody, showing that TGF-β1 neutralization in HBMSCs reduced the effect of anti-myofibroblast differentiation on CTD-UIP HLFs (Fig. 7d). This may explain why HBMSCs expressing TGF-β1 have an antifibrotic capability.

### Supernatants harvested from HBMSCs can improve the survival rate in BLM-induced pulmonary fibrosis mice

**Fig. 8:**
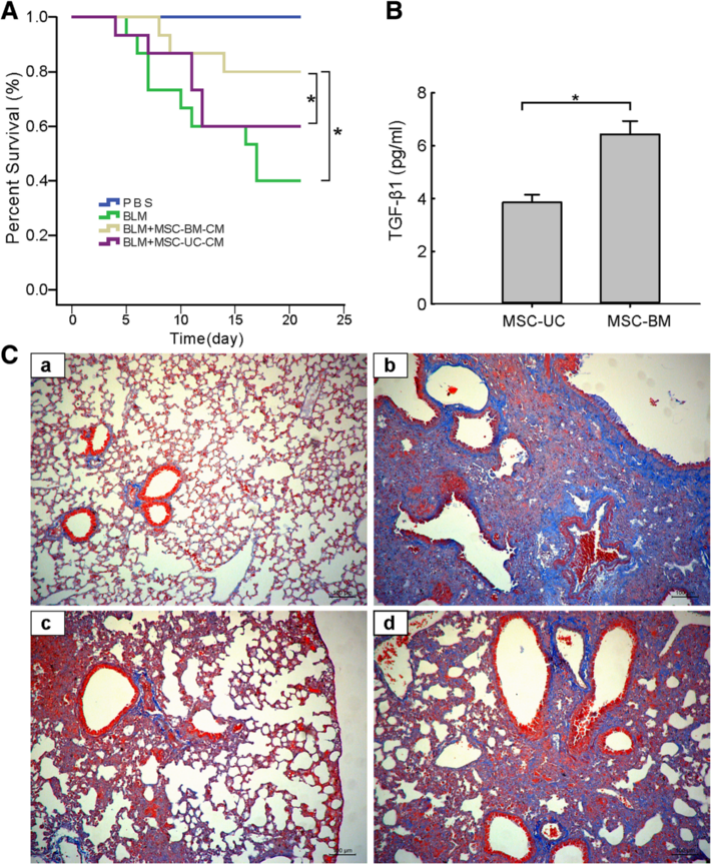
Mesenchymal stem cells from bone marrow and umbilical cord exert different efficacy in BLM-induced pulmonary fibrosis mouse model. (A) Survival rates of C57BL/6 mice in the control group and BLM-induced group without any treatment or with treatment by supernatant from either MSCs-BM or MSCs-UC. Supernatants harvested from MSC (1 × 10^6^) culture were intratracheally administered to mice 48 hours after BLM treatment. Analysis was conducted by a logrank test based on the Kaplan–Meier method. (B) An enzyme-linked immunosorbent assay demonstrated a significantly higher level of TGF-β1 secreted from HBMSCs than from MSC-UC. (C) Representative Masson staining photomicrographs of the lung tissue sections from mice 21 days after saline exposure (a), BLM exposure (b), BLM exposure with treatment of the supernatant from MSC-BM (c), and BLM exposure with treatment of the supernatant from MSC-UC (d). 200× magnification. *MSCs-BM* mesenchymal stem cells isolated from bone marrow, MSCs-UC mesenchymal stem cells isolated from umbilical cord, *TGF-β1* transforming growth factor-β1

Finally, we evaluated the antifibrotic efficacy of TGFβ1-hypersecreting HBMSCs in a BLM-induced pulmonary fibrosis mouse model. By making a comparison of antifibrotic capability in supernatants between TGFβ1-high and TGFβ1-low, derived from MSCs originated from different sources, we demonstrate that the supernatant derived from HBMSCs expressing a high level of TGFβ1 has a better therapeutic efficacy on improving the survival rate, as well as reducing pulmonary inflammation and fibrosis than that from MSCs-UC which secrete a lower level of TGFβ1 (Fig. 8).

## Discussion

In the present study, we first reported that persistent activation of natural killer T cells (NKTs) is accompanied by attenuation or deficiency of the regulatory T cell (Treg) response in interstitial pneumonia in connective tissue diseases (CTD-IP). We further disclosed the proinflammatory and profibrotic properties of lung fibroblasts in CTD-IP patients pathologically diagnosed with UIP. To the best of our knowledge, this study is the first to reveal that HBMSCs with a high level of TGF-β1 secretion can redress aberrant TGF-β1 downstream signal transduction for regulation of excessive IL-6/STAT3 signaling, consequent to Treg expansion, and to induction of anti-fibrotic cytokine expression.

NKT cells, a heterogeneous group of T lymphocytes, are known to functionally bridge the innate and adaptive immune system in various immune diseases due to their cytotoxic function and production of the proinflammatory factors IL-4 and IFN-γ [27]. A recent study showed that IFN-γ-producing NKT cells promoted immune complex (IC)-induced acute lung injury by stimulating production of MIP-1 through both autocrine and paracrine mechanisms, and by enhancing cytokine production from alveolar macrophages and CD11c^+^ dendritic cells (DCs) [28]. In the present study, we found that CTD-IP patients with active disease had a higher frequency of NKTs in their peripheral blood and lungs, where the disruption of the normal alveolar architecture was accompanied by patchy alveolar pneumocyte hyperplasia and fibrosing changes. Therefore, uncontrolled activation of a NKT cell-mediated abnormal immune response could contribute to chronic lung injury, inflammation and abnormal repair with diffuse fibrosis in CTD-IP patients. Among T cells subsets, Tregs have a known role in controlling overt inflammation [29]. A systemic defect in Tregs is linked to inferior lung function in the enrolled CTD-IP patients, which is parallel to that observed in patients with idiopathic pulmonary fibrosis (IPF) [9], suggesting that pulmonary fibrotic progression in IPF and CTD-IP patients is associated with the failure of inflammation resolution due to deficiency of Treg manipulation.

A number of investigations have provided convincing evidence showing that interstitial fibroblasts in an inflammatory microenvironment produced by cytotoxic T cell recruitment to lung, are activated and differentiate towards a myofibroblast phenotype [30]. We detected the myofibroblast phenotypes in pulmonary fibroblasts isolated from CTD-UIP lungs, where cytokine/chemokine profiles are characterized with a remarkable increase in IL-6 secretion accompanied by chemokine up-regulation, indicating that the abnormal lung interstitial fibroblasts may disturb Treg differentiation whereby cytotoxic immune cells, such as NKT and CD8^+^ T cells, maintain activation in lung parenchyma. This may create an uncontrolled positive feedback loop for immune activation and inflammation, which will make conventional anti-inflammatory therapy ineffective in the management of CTD-IP. Breaking this feedback loop so as to restore a normal balance between different subsets of immune cells, rather than using indiscriminate anti-inflammatory agents, may be a promising approach for treating CTD-IP [9, 31].

Many studies reported that MSC-mediated cell therapy is very effective in treating autoimmune diseases [17–19]. We show that HBMSCs induce Treg proliferation in an experimental NKT-PBMC model in vitro, whereas high frequencies of NKT and CD8^+^ T cells are reduced. Importantly, we found that the HBMSCs self-secreting a high level of TGF-β1 can facilitate the growth of Tregs in PBMCs isolated from IPF patients as well. These results indicate that MSC-based therapy may allow repair of impaired Tregs through a TGF-β1-dependent regulation, by which cytotoxic T cells are suppressed, rather than by universally inhibiting T cells proliferation.

There is increasing evidence showing that MSCs exert immunosuppressive effects on immune inflammation through the release of many soluble cytokines including TGF-β1, PGE2, indoleamine 2, 3-dioxygenase (IDO), IL-10, and IL-1RA [17, 32–34]. A prominent function of TGF-β1 is regulating immune homeostasis and TGF-β1 deficiency in mice results in excessive inflammation and lethality [35]. Abnormally activated T cells and elevated proinflammatory cytokines, including TNF-α, IFN-γ, and IL-1β, have been detected in TGF-β1 knockout mice [36]. Moreover, endogenous TGF-β1 is essential for the induction of immunosuppressive Treg cells [37, 38]. However, we show a significant up-regulation of the TGF-β1 level accompanied by a reduced Tregs and down-regulation of the ratio of TGF-β1 to IL-6 in the CTD-IP patients, reflecting that the increase of endogenous TGF-β1 released from immunocytes in response to the inflammatory microenvironment could not induce Tregs differentiation owing to IL-6 hypersecretion that causes an imbalance between IL-6 and TGF-β1 in local and systemic modulation of the immune response, thereby disturbing TGF-β1 signaling. A high level of TGF-β1 self-secretion by HBMSCs may therefore be an important mechanism underlying therapeutic effects of MSCs on promoting Tregs expansion in IPF patients [39, 40].

TGF-β signaling is also involved in normal lung development and injury repair [41, 42]. On the contrary, it is able to induce fibroblast proliferation, differentiation, migration, and extracellular matrix production and contraction. In the adult lung, excessive TGF-β-mediated Smad3 signaling, as seen after bleomycin administration, plays a critical role in extensive fibrosis [43]. The current study demonstrates an excessive IL-6 secretion and substantially reduced IP-10 expression, but neither a high level of TGF-β1 nor activated TGF-β-mediated Smad3 signaling in CTD-UIP-HLFs which represent a myofibroblast phenotype. Overproduction of the IL-6 family of cytokines, aberrant activation of their receptors or receptor-associated tyrosine kinases, or epigenetic alterations or mutations in genes encoding negative regulators of STAT3 can bring about persistent STAT3 activation [44–46]. Elevated tyrosine phosphorylation of STAT3 is able to suppress apoptosis and promote angiogenesis and fibrotic proliferation [44]. It has been reported that TGF-β-mediated biological responses are impaired in mice in which STAT3 is excessively activated due to its upstream receptor gp130 mutation. Activated STAT3 in turn elicits the increased expression of the TGF-β signaling inhibitory molecule Smad7, thereby inhibiting the intracellular activity of TGF-β signaling [47].

In general, TGF-β1 can stimulate fibroblast differentiation to the myofibroblast phenotype and suppress myofibroblast apoptosis [48]. However, we show that either HBMSCs self-secreting a high level of TGF-β1 or TGF-β1 added to CTD-UIP-HLFs can induce production of anti-fibrotic chemokine IP-10 [49–52], which may act downstream of TGF-β signaling to negatively regulate activation of myofibroblasts marker [53], leading to attenuation of α-SMA over expression in the treated CTD-UIP-HLFs.

IP-10 is up-regulated after both immune and non-immune mediated tissue injury but is an antifibrotic chemokine involved in tissue repair and remodeling [49, 50, 54]. We and other investigators have found downregulation of IP-10 expression in fibroblasts isolated from CTD-IP (pathologically diagnosed UIP) and IPF lungs, which contributes to the myofibroblast phenotype [55, 56]. Although the ability to inhibit fibroblast migration is thought to be an important mechanism of IP-10 in limiting the development of fibrosis [49, 54], the effect of IP-10 on α-SMA expression in CTD-UIP-HLFs is still unclear. We, for the first time, demonstrate that TGF-β1 released from MSCs may block myofibroblast activation in CTD-UIP HLFs through sensitizing the TGFβ/Smad signaling pathway that is severely attenuated by excessive IL-6/STAT3 signaling, thereby overcoming the proinflammatory phenotype and relieving the inhibition of IP-10 expression to push against myofibroblast differentiation.

The present study reveals that in patients with CTD-IP, high levels of IL-6 secretion are predominantly associated with pulmonary fibrotic progression. A similar finding reported by Collard and Alhamad has been shown in IPF patients with acute exacerbation [57, 58]. A phase 1b study of placenta-derived mesenchymal stromal cells in IPF patients has recently demonstrated that intravenous MSC administration is feasible and has a good short-term safety profile in patients with moderately severe IPF [59]. Herein we provide, for the first time, clear evidence in vivo showing that MSCs with a higher level of TGFβ1 self-secretion may have an optimal therapeutic efficacy on the counteraction of life-threatening pulmonary fibrotic exacerbation.

## Conclusions

Our study provides the first evidence that persistent activation of cytotoxic immune cells, particularly NKTs, accompanied by attenuation or deficiency in Tregs relevant to IL-6 hyper-induction, strongly correlate with fibrotic exacerbation in CTD-IP. MSC-based cell therapy appears to be a promising approach for treating pulmonary fibrotic progression in CTD-IP, the underlying mechanism for which is attributable, at least in part, to the characterization of TGF-β1 hyper-secretion in HBMSCs. This is linked to activation of impaired TGF-β downstream signal pathway, thereby regulating excessive IL-6/STAT3, whereby a relief of suppression in Tregs differentiation and expansion may concomitantly activate anti-fibrotic IP-10 expression. This may in turn block progression of lung fibrosis.

## Additional files

Additional file 1: Supplementary material online. Methods. (DOC 79 kb)

Additional file 2: Figure S1. The biological characteristics of human bone marrow MSCs. Flow cytometric analysis at passage 4–6 demonstrated that MSCs were negative for CD14, CD34, CD45, and CD11a, but were positive for CD105, CD90, CD44, and CD29. (TIF 242 kb)

Additional file 3: Figure S2. Cell immunophenotypes of human induced NKT-PBMCs. Cell phenotypes in the peripheral blood of the healthy volunteer and NKT-PBMCs cultures on day 14 were observed. (**A**), (**B**), (**C**), and (**D**) show the dot plots and summary data of flow cytometric analysis. After the 14-day culture period, a higher frequency of CD3^+^CD8^+^ T cells (**C**), CD3^+^CD56^+^ NKT cells (**D**) and a lower frequency of CD3^+^CD4^+^ T cells (**B**) were observed. The mean ± SD of five cases in each group are shown in (**B**), (**C**), and (**D**). ** *P* < 0.01. (TIF 481 kb)

Additional file 4: Figure S3. Immunomodulatory effects of human bone marrow MSCs on NKT-PBMCs. NKT-PBMCs were co-cultured with human MSCs or human fibroblasts at a 20:1 ratio of NKT-PBMCs to human MSCs or NHLF prior to flow cytometric analysis. Flow cytometric analysis of CD3^+^ CD56^+^ cells, CD3^+^ CD8^+^ cells, CD3^+^ CD4^+^ cells gating on CD45^+^ cells, and CD25^+^ CD127^(Low/-)^ cells gating on CD4^+^ cells, of either NKT-PBMCs (NKT-PBMCs), or NKT- PBMCs co-cultured with human bone MSCs (NKT-PBMCs/MSC), or co-cultured with NHLF (NKT-PBMCs/NHLF). (TIF 2388 kb)

## Abbreviations

BAL: bronchoalveolar lavage
CTD-IP: interstitial pneumonia in connective tissue diseases
CTD-UIP-HLF: HLF isolated from the lung tissues pathologically diagnosed with UIP in CTD-IP patients
FVC: forced vital capacity
H&E: hematoxylin and eosin
HBMSCs: human bone marrow mesenchymal stem cells
HLFs: human primary lung fibroblasts
IDO: indoleamine 2, 3-dioxygenase
IFN-γ: interferon γ
IL-1β: interleukin 1 beta
IP: interstitial pneumonia
IP-10: interferon γ-induced protein 10
IPF: idiopathic pulmonary fibrosis
MSC-BM: MSCs isolated from bone marrow (BM)
MSCs: mesenchymal stem cells
MSC-UC: MSCs isolated from umbilical cord (UC)
NKTs: natural killer T cells
PBMCs: peripheral blood mononuclear cells
PM/DM: polymyositis/dermatomyositis
RA: rheumatoid arthritis
STAT3: signal transducer and activator of transcription 3
TGF-β: transforming growth factor-β
TNF-α: tumor necrosis factor α
Tregs: regulatory T cells
UIP: usual interstitial pneumonia
VATS: video-assisted thoracoscopic surgery
α-SMA: α-smooth muscle actin
