# Supplementary material for: Immunomodulation by mesenchymal stem cells in treating human autoimmune disease-associated lung fibrosis
Source: Stem Cell Res Ther. 2016 Apr 23;7:63. doi: 10.1186/s13287-016-0319-y (PMC4842299; doi:10.1186/s13287-016-0319-y)
Supplement: Supplementary file 1 — Supplementary material online. Methods. (DOC 79 kb) [file 13287_2016_319_MOESM1_ESM.doc]

**Supplementary material online**

**Culture and identification of human bone marrow mesenchymal stem cells**

Human bone marrow mesenchymal stem cells (HBMSCs) were isolated from the bone marrow of normal individuals undergoing bone marrow harvest for allogeneic bone marrow transplantation, according to institutional guidelines as previously described. Briefly, mononuclear cells were separated by centrifugation over a Ficoll-Hypaque gradient and suspended in MSC complete medium: LG-DMEM medium containing 100 IU/ml penicillin, 100 µg/ml streptomycin, L-glutamine and 20% fetal bovine serum (FBS) (Gibco/Invitrogen, Carlsbad, CA), and plated in 180 cm2 plastic dish. After 3 days, the non-adherent cells were removed by washing with phosphate buffered saline (PBS) and monolayers of adherent cells were cultured until they reached confluence. Cells were then trypsinized (0.25% trypsin with 0.02% EDTA) and sub-cultured at densities of 5,000-6,000 cells/cm^2^. Identified cell phenotypes, then cells were passaged by regular 1:3 subculture, and cells between 5-10 passages were used for the experiments. The morphological characteristic and immunological features of HBMSCs were shown in figure E1.

**Generation and identification of human induced NKT-PBMCs (natural killer T cells-peripheral blood mononuclear cells)**

Peripheral blood (PB) of blood donors were provided by the blood center in Guangzhou, which was approved by the Guangdong provincial health department. Ficoll-Hypaque density gradient centrifugation was performed for collecting PBMCs. On day 0, the cells (2 × 10^6^ cells/ml) were cultured using fresh serum-free AIM-V medium (GIBCO, U.S.A) with stimulation of recombinant human IFN-γ (1,000 IU/ml, CLONGAMMA, CN). The following day, αCD3 mAb (50ng/ml, R&D, U.S.A) and recombinant human IL-2 (rHuIL-2, 300 IU/ml, SHUANGLU, CN) were added into the culture. Cell suspensions were maintained in subculture with fresh medium supplemented with rHuIL-2 every 3 days for 2 weeks. Cells phenotypes were observed by flow cytometry (Flow cytometry, Beckman, USA) (figure E2), and were collected on day 14 for following experiments.

**Culture of primary human lung fibroblasts**

Primary Human lung fibroblasts (HLFs) from normal human lung and patients with CTD-IP were acquired under a procedure defined by the University of Iowa Institutional Review Board (Iowa City, IA), which was performed as previously described. Briefly, lung tissues were obtained at the time of biopsy using video-assisted thoracoscopic surgery (VATS) following procedures approved by the Ethics Committee of the First Affiliated Hospital of Guangzhou Medical University. After washing with PBS, lung tissue explants were cut into several small pieces and incubated in tissue culture dishes in complete culture medium (DMEM-F12, 10%FBS, and 1% penicillin/streptomycin) at 37°C with 5% CO2. Then changed the medium every 3 days and outgrowth was evident in 5-7 days. Upon reaching 80% confluency, the tissue pieces were removed and the cells were trypsinised and seeded for subsequent cell passages. Fibroblasts were used between passages 3 to 5 in this study. Cells were characterized as typical spindle morphology, were positive staining with vimentin and α smooth muscle actin. **Cytotoxicity assay**

The cytotoxicity assay of NKT-PBMCs against human pulmonary epithelial cell line (16HBE) was measured using Cell Counting Kit-8 (Dojindo, Kumamoto, Japan), 100ul of 16HBE suspension (5,000 cells) was added into per well of 96-well plate, and incubated for 8 hours at 37°C, then either exposed or not-exposed to 100ul of NKT-PBMCs (100,000 cells), after 4-h incubation at 37°C, 20ul of CCK-8 solution were added into per well. Plates were incubated at 37°C for 2.5 h; the absorbance was measured by Spectra Max M5 (Molecular Devices, USA) at 450 nm. The cytotoxicity (%) was calculated using the following formula: 1 − 100% × [OD (experiment)–OD (NKT-PBMCs)/OD (control) −OD (blank)]. OD (experiment) refers to the absorbance of a well with 16HBE cells, NKT-PBMCs, and CCK-8, OD (NKT-PBMCs) is the absorbance of a well well with NKT-PBMCs and CCK-8, while OD (blank) is the absorbance of a well with medium and CCK-8 but without cells, and OD (control) is the absorbance of16HBE cells and CCK-8.

**References**

1. Berman DM, Willman MA, Han D, Kleiner G, Kenyon NM, Cabrera O et al. Mesenchymal stem cells enhance allogeneic islet engraftment in nonhuman primates. Diabetes. 2010;59(10):2558-68. doi:10.2337/db10-0136.

2. Nyunoya T, Monick MM, Klingelhutz AL, Glaser H, Cagley JR, Brown CO et al. Cigarette smoke induces cellular senescence via Werner's syndrome protein down-regulation. American journal of respiratory and critical care medicine. 2009;179(4):279-87. doi:10.1164/rccm.200802-320OC.
