# Supplementary figures and images for: Immunomodulation by mesenchymal stem cells in treating human autoimmune disease-associated lung fibrosis
Source: Stem Cell Res Ther. 2016 Apr 23;7:63. doi: 10.1186/s13287-016-0319-y (PMC4842299; doi:10.1186/s13287-016-0319-y)

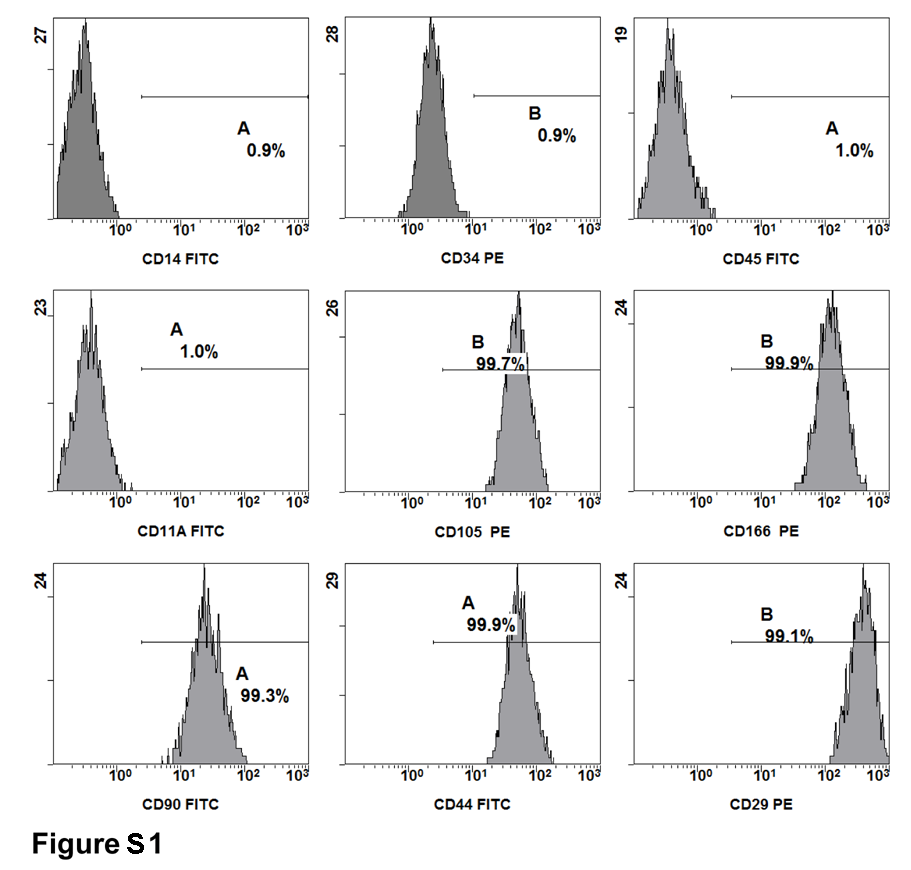

Supplement: Supplementary file 2 — The biological characteristics of human bone marrow MSCs. Flow cytometric analysis at passage 4–6 demonstrated that MSCs were negative for CD14, CD34, CD45, and CD11a, but were positive for CD105, CD90, CD44, and CD29. (TIF 242 kb) [file 13287_2016_319_MOESM2_ESM.tif]

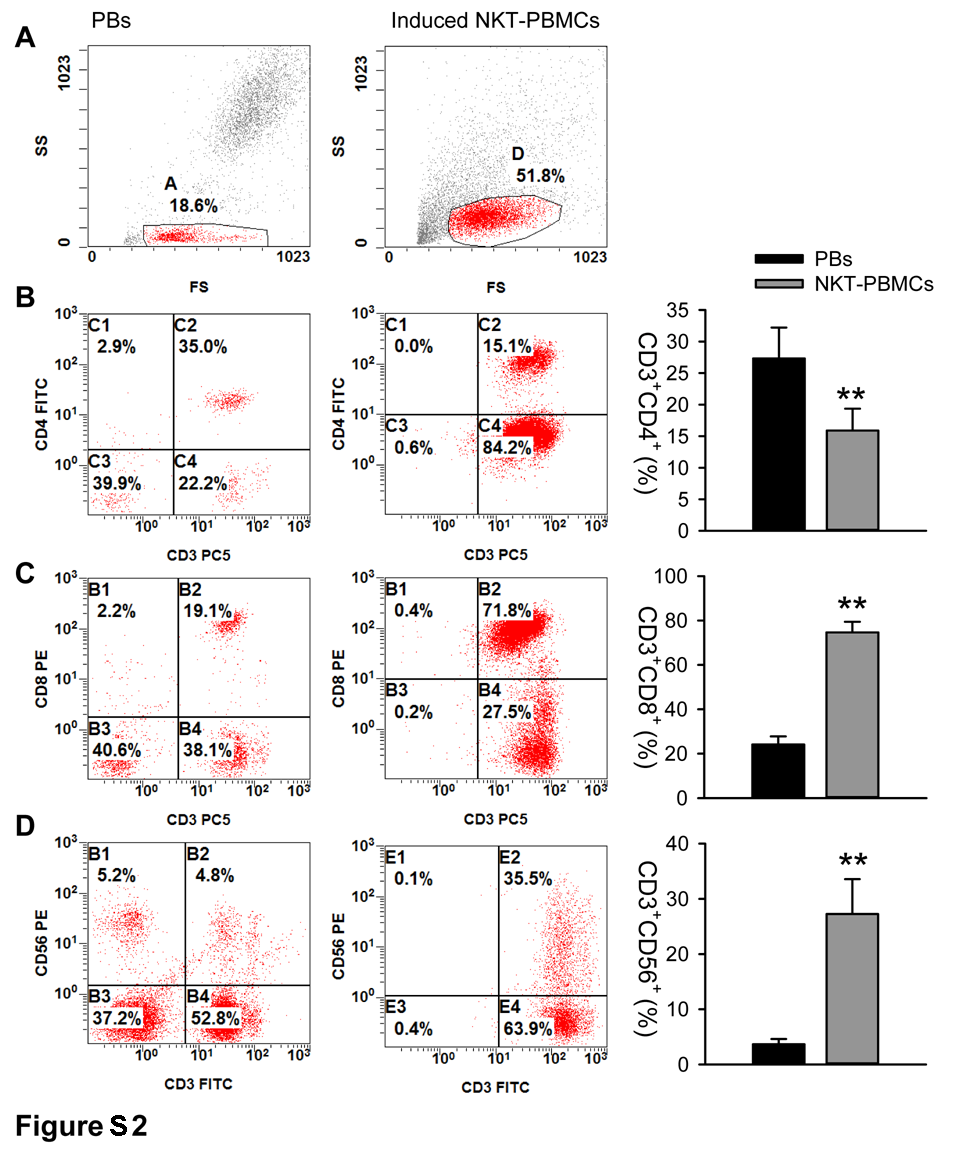

Supplement: Supplementary file 3 — Cell immunophenotypes of human induced NKT-PBMCs. Cell phenotypes in the peripheral blood of the healthy volunteer and NKT-PBMCs cultures on day 14 were observed. (A), (B), (C), and (D) show the dot plots and summary data of flow cytometric analysis. After the 14-day culture period, a higher frequency of CD3+CD8+ T cells (C), CD3+CD56+ NKT cells (D) and a lower frequency of CD3+CD4+ T cells (B) were observed. The mean ± SD of five cases in each group are shown in (B), (C), and (D). ** P < 0.01. (TIF 481 kb) [file 13287_2016_319_MOESM3_ESM.tif]

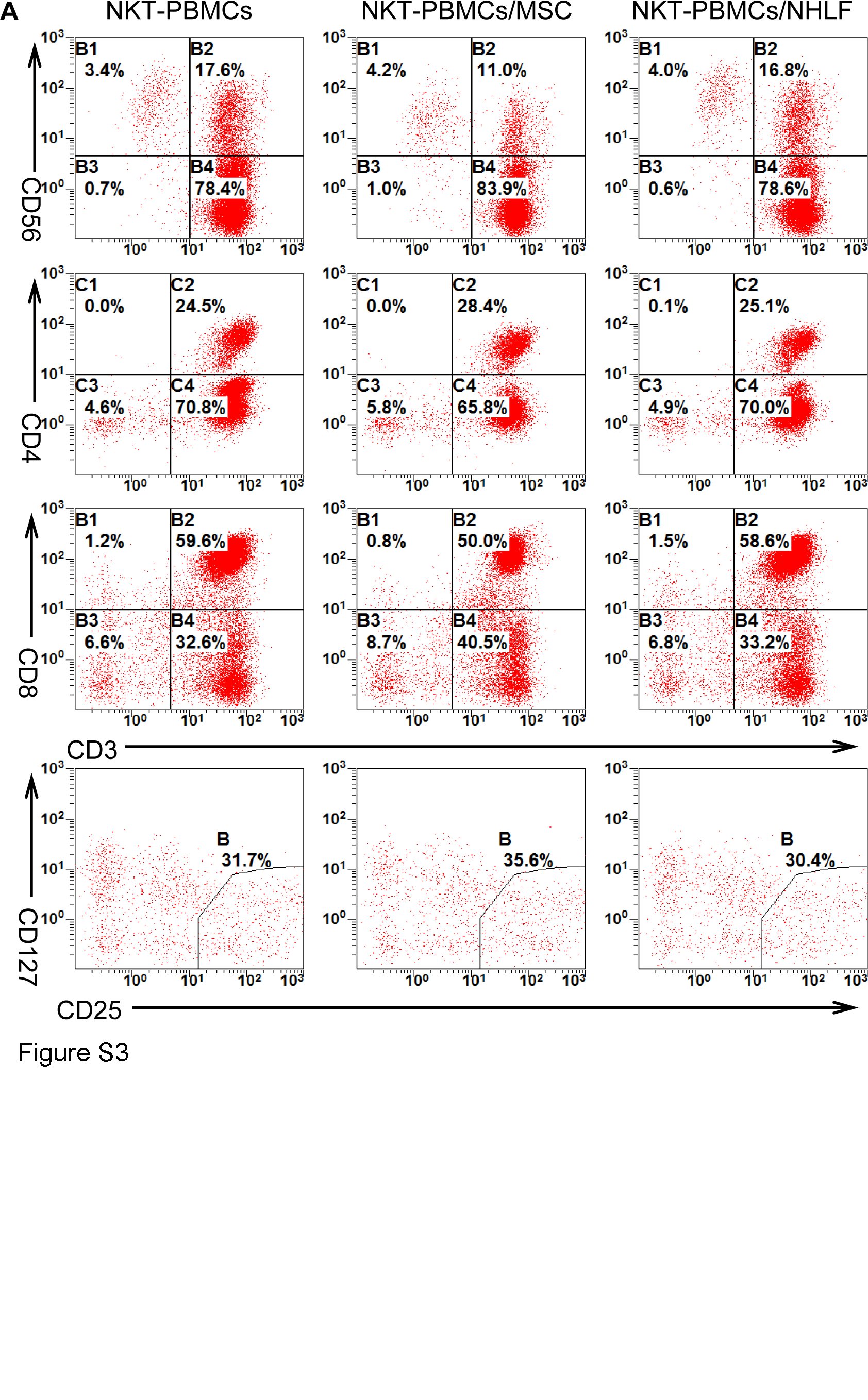

Supplement: Supplementary file 4 — Immunomodulatory effects of human bone marrow MSCs on NKT-PBMCs. NKT-PBMCs were co-cultured with human MSCs or human fibroblasts at a 20:1 ratio of NKT-PBMCs to human MSCs or NHLF prior to flow cytometric analysis. Flow cytometric analysis of CD3+ CD56+ cells, CD3+ CD8+ cells, CD3+ CD4+ cells gating on CD45+ cells, and CD25+ CD127(Low/-) cells gating on CD4+ cells, of either NKT-PBMCs (NKT-PBMCs), or NKT- PBMCs co-cultured with human bone MSCs (NKT-PBMCs/MSC), or co-cultured with NHLF (NKT-PBMCs/NHLF). (TIF 2388 kb) [file 13287_2016_319_MOESM4_ESM.tif]
